# Estimates of wind power and radiative near-inertial internal wave flux: The hybrid slab model and its application to the North Atlantic

**DOI:** 10.1007/s10236-020-01388-y

**Published:** 2020-09-09

**Authors:** Georg S. Voelker, Dirk Olbers, Maren Walter, Christian Mertens, Paul G. Myers

**Affiliations:** 1 MARUM - Center for Marine Environmental Sciences, University of Bremen, Bremen, Germany; 2 Alfred Wegener Institute for Polar and Marine Research, Bremerhaven, Germany; 3 Institute of Environmental Physics, University of Bremen, Bremen, Germany; 4 Department of Earth and Atmospheric Sciences, University of Alberta, Edmonton, Alberta Canada

**Keywords:** Near inertial waves, Wind ocean coupling, Internal gravity waves

## Abstract

Energy transfer mechanisms between the atmosphere and the deep ocean have been studied for many years. Their importance to the ocean’s energy balance and possible implications on mixing are widely accepted. The slab model by Pollard (Deep-Sea Res Oceanogr Abstr 17(4):795–812, 1970) is a well-established simulation of near-inertial motion and energy inferred through wind-ocean interaction. Such a model is set up with hourly wind forcing from the NCEP-CFSR reanalysis that allows computations up to high latitudes without loss of resonance. Augmenting the one-dimensional model with the horizontal divergence of the near-inertial current field leads to direct estimates of energy transfer spectra of internal wave radiation from the mixed layer base into the ocean interior. Calculations using this hybrid model are carried out for the North Atlantic during the years 1989 and 1996, which are associated with positive and negative North Atlantic Oscillation index, respectively. Results indicate a range of meridional regimes with distinct energy transfer ratios. These are interpreted in terms of the mixed layer depth, the buoyancy frequency at the mixed layer base, and the wind field structure. The average ratio of radiated energy fluxes from the mixed layer to near-inertial wind power for both years is approximately 12%. The dependence on the wind structure is supported by simulations of idealized wind stress fronts with variable width and translation speeds.

## Introduction

The excitation of near-inertial internal gravity waves by wind stress and the corresponding energy transfer mechanisms have been studied for decades. Many experiments were conducted in the 1970s and 1980s (e.g., Kundu 1976; Pollard 1980; Fu 1981; Price 1983; D’Asaro 1985) followed by the work of D’Asaro et al. (1995). These studies linked the velocity variability below the mixed layer to the wind field and found near-inertial components to be remarkably dominant. In particular, most observations showed upward propagation of phase and thus downward propagation of energy (Leaman and Sanford 1975; Leaman 1976). At the same time, a range of models were developed to understand the corresponding excitation mechanisms (e.g., Pollard and Millard 1970; Käse and Olbers 1980; D’Asaro^1985^; Price et al. 1986). Recently, the energy transfer from the wind to the surface ocean, as well as the energy budgets of the mixed layer and the wave radiation below, has been investigated using an extension of the Ekman spiral (Kim et al. 2014), extensions of the slab model introduced by Pollard and Millard (1970), and high-resolution ocean models (e.g., Furuichi et al. 2008; Simmons and Alford 2012; Rimac et al.^2013^).

In particular, the slab model has drawn a lot of attention in a range of contexts. The first version (Pollard and Millard 1970) was established to interpret moored velocity records and link near-inertial fluctuations to wind-driven momentum fluxes (e.g., Paduan et al. 1989; Pollard 1980). D’Asaro (1985) extended the model by subtracting a time-dependent Ekman component to isolate the inertial response. Taking into account the spectral solution method suggested by Alford (2001), this formulation is the most applied to date. Its use ranges from comparison with and interpretation of local measurements (e.g., Alford 2001; Plueddemann and Farrar 2006; Chaigneau et al. 2008) to global estimates of near-inertial wind power input (e.g., Alford 2001; Watanabe and Hibiya 2002; Furuichi et al. 2008). The formulation of a frequency-dependent attenuation of the induced surface motion has received little attention so far (Alford 2003; Alford et al. 2012). Whitt and Thomas (2015) and Jing et al. (2017) extended the slab model of Pollard and Millard (1970) by an underlying geostrophic motion and showed that the geostrophic flow may affect the near-inertial wind power.

Directly related is the question of how the wind-induced near-inertial motion near the surface generates internal gravity waves and propagates into the deep ocean. Gill (1984) suggested that the divergence of a wind-induced near-inertial oscillation may generate vertical velocities which in return may propagate downward as linear internal gravity waves with the corresponding dispersion relation and group velocities. However, D’Asaro (1984) noted that the group velocities estimated from the observed properties of the radiated internal gravity waves were small and did not fully account for the observed rate of energy transmission. Following their analysis, it has been shown extensively that a spatially varying vertical relative vorticity associated to a geostrophic mean-flow may modulate the generated wave field such that the vertical propagation is enhanced significantly (Kunze 1985; Young and Ben Jelloul 1997; Balmforth et al. 1998; Zhai et al. 2005; Jing and Wu 2014).

This study uses the slab model of Pollard and Millard (1970) and adds an extension introduced in Olbers et al. (2012). In particular, “inertial pumping” velocities (cf. Gill, 1984 ), i.e., vertical velocities at the base of the mixed layer, are considered a boundary condition to a linear internal gravity wave field being a disturbance to an ocean at rest with a locally constant buoyancy frequency. The approach does thus not include wave modulation, which is a known mechanism for enhancing vertical propagation of wave energy as explained above. The vertical pumping velocities are derived from a grid of pointwise-calculated slab model results. Their lateral structure and magnitude are combined with the local buoyancy frequency at the mixed layer base to obtain an estimate of the radiation energy flux from the mixed layer base into the deep ocean. This approach allows us to quantify the radiation energy flux from the mixed layer into the internal wave field of the stratified ocean below. However, assumptions on the horizontal wave number spectrum as well as the local buoyancy frequency at the mixed layer base must be made. We refer to this extended slab model as the hybrid slab model.

This paper presents internal wave energy fluxes derived from simulations of the hybrid slab model tuned to represent the North Atlantic during the years 1989 and 1996. Even though the North Atlantic covers only a relatively small part of the world’s ocean, it is affected by atmospheric forcing with a broad range of temporal and spatial scales. Many different atmospheric forcing features are observed, including tropical trade winds, hurricanes, and mid-latitude synoptic-scale storms. In addition, deep convection occurs at high latitudes. The chosen years represent two opposite phases of the North Atlantic Oscillation (NAO). Whereas 1989 is characterized by a positive NAO phase with relatively strong winds in the mid-latitudes, 1996 is an example of a negative NAO phase with relatively weak winds in the same region (Hurrell and Deser 2009). The physical mechanisms driving the spatial and temporal variations in the flux of energy into the internal wave field in these two realistic simulations are explored using theory and idealized simulations.

An extended slab model based on the same ideas as presented in this study is presented in a companion paper by Olbers et al. (2020). They derive an extended slab model which includes wave radiation through taking into account the local pressure perturbation and compare the resulting energy transfer spectra to the hybrid slab model. A first application of the hybrid slab model with qualitative comparisons to near-inertial energy levels in spectra from moored records was presented by Köhler et al. (2018).

The outline of the paper is as follows: First, we recall the mathematical formulation of Pollard and Millard (1970) and introduce the hybrid extension (Section 2). Then, we discuss the input data driving our model (Section 3). Subsequently, results for simulations over the North Atlantic for the years 1989 and 1996 are presented (Section 4). We then conclude with a discussion focusing on the input parameter dependencies and structural patterns in the wind field taking idealized simulations into account (Section 5).

## Description of slab and hybrid models

### The slab model

Since introduced by Pollard and Millard (1970) and revisited by D’Asaro (1985), the slab model equations have been widely used to estimate the power transferred from the wind to the ocean surface mixed layer. They assume a homogeneous slab of depth *D* with constant density *ρ* and an internal stress that is equal to the wind stress, *τ*_0_, on the surface and zero at the base of the mixed layer. Since the equation of motion is integrated in the vertical, the profile of the internal stresses is arbitrary and does not need to be prescribed. The physics are reduced to the corresponding wind stress term, the Coriolis effect, and a damping term. The latter parameterizes the sinks of kinetic energy in the mixed layer such as turbulent dissipation, entrainment, or radiation of internal waves at the mixed layer base. Integrated over the mixed layer, the governing equation reads in complex form:

1 $$ {\frac{\partial}{\partial t}} (u + iv) + (if + r)(u + iv) = \xi . $$

where (*u* + *iv*) are the depth averaged horizontal velocity components in the mixed layer, *f* is the Coriolis frequency, and *r* represents a damping parameter. The source term *ξ*(***x***, *t*) = *ξ*_*x*_ + *iξ*_*y*_ = *τ*_0_(***x***, *t*)/*ρD* contains the constant density *ρ* = 1027kg m^− 3^, the time-dependent mixed layer depth *D*(*t*), and wind stress components, *τ*_0_ = *τ*_0*x*_ + *iτ*_0*y*_. The Rayleigh damping term, *r*(*u* + *iv*), parameterizes all dissipation mechanisms, including turbulent dissipation, mixed layer entrainment, and internal wave generation. Moreover, it is assumed that the horizontal velocity, (*u* + *iv*), is approximately uniform throughout the mixed layer and the mixed layer depth, *D*, varies more slowly than the horizontal mixed layer velocities so that $\partial /\partial t \left [D(u + iv)\right ] \approx D \partial /\partial t (u + iv)$.

We solve the slab mixed layer equation in Fourier space. In particular, taking the transformation of Eq. 1 in time and the horizontal yields:

2 $$ \left[i(\sigma + f) + r\right] (\hat{u} + iv) = \hat{\xi}, $$

with Fourier transform:

3 $$ \hat{u}(\boldsymbol{k}, \sigma) = \underset{\mathbb{R}^{3}}{\iiint} u(\boldsymbol{x}, t)  e^{-i(\boldsymbol{k} \cdot \boldsymbol{x} + \sigma t)}  d\boldsymbol{x} dt. $$

where *σ* represents the angular frequency. Note that the spatial Fourier transform is made here for later use in the hybrid slab model. Now, the equation can readily be solved as follows:

4 $$ \hat{u} + i\hat{v} = \mathsf{T} \hat{\xi} = \left[i(\sigma + f) + r\right]^{-1} \hat{\xi}. $$

The solution given by Eq. 4 also includes the well-known Ekman solution to the constant component of the wind stress. By definition, the Ekman response is constant in time and is therefore equivalent to the zero frequency response. Consequently, it has no effect on excitation of internal waves with frequencies between the inertial and buoyancy frequencies. For reasons of consistency, no distinction between near-inertial and Ekman components is made. The horizontal slab velocities are then evaluated taking the inverse Fourier transform of Eq. 4.

We evaluate the wind power by considering the kinetic energy balance of the surface slab. The total energy budget is obtained by multiplying (1) with $(\hat {u} - i\hat {v})$ and taking the real part. In particular:

5 $$ {\frac{\partial}{\partial t}} \left( D \frac{\rho}{2} (u^{2} + v^{2}) \right) = (\tau_{0x}u + \tau_{0y}v) - r  (\rho D)  (u^{2} + v^{2}). $$

Whereas the term − *r*(*ρD*)(*u*^2^ + *v*^2^) describes the energy loss associated to the Rayleigh damping, the first term describes the total wind power:

6 $$ \phi(t) = (\tau_{0x}u + \tau_{0y}v). $$

Note that the wind power, *ϕ*, is also commonly noted in vector form so that *ϕ* = ***τ***_0_ ⋅***u***. The slab model velocities (4) resonate at the frequency *σ* = −*f*. Motivated by this resonance as well as the fact that sub-inertial frequencies which dominate the slab response in a typically pink to red wind stress spectrum (Gille 2005) but cannot contribute to the wave generation we band pass filter the slab response using an order-1 Butterworth filter within the frequency range [0.7*f*,1.3*f*]. The filtered velocities are then denoted by $u_{_{\text {NI}}}$ and $v_{_{\text {NI}}}$. The corresponding near-inertial wind power, $\phi _{_{\text {NI}}}$, is defined as:

7 $$ \phi_{_{\text{NI}}}(t) = (\tau_{0x}u_{_{\text{NI}}} + \tau_{0y}v_{_{\text{NI}}}). $$

As the near-inertial wind power depends on the damping parameter, *r*, the latter must be chosen with care. A detailed review of the parameter dependencies and the resonance of the slab model appears in Appendix A.

### The hybrid solution

According to Eq. 4, the average mixed layer velocity depends on the damping parameter, Coriolis frequency, mixed layer depth, and wind stress. Consequently, the horizontal divergence of the field is generally non-zero. The continuity equation, integrated from the mixed layer depth *D* to the surface, yields:

8 $$  \left. w \right|_{z=-D} = D  \boldsymbol{\nabla} \cdot \boldsymbol{u} $$

Here, the rigid lid approximation is used. Consequently, any divergent near-inertial surface velocity field, ***u***, induces “inertial pumping” (cf. Gill, 1984). These vertical oscillations can be considered as boundary condition to a stratified layer below the mixed layer base. As we are interested in the downward radiation of the slab-layer wave-like motion in the form of internal gravity waves, we consider a linear WKBJ field being a disturbance to an ocean at rest. Hence, this approach neglects wave modulation by a sheared background flow as well as wave-mean flow interactions. An intensification of internal gravity wave radiation based on increased vertical group velocities due to wave modulation as previously reported (Kunze 1985; Young and Ben Jelloul 1997; Balmforth et al. 1998; Zhai et al. 2005; Jing and Wu 2014; Whitt and Thomas 2015; Jing et al. 2017) is not included here. Moreover, we assume a locally constant (i.e., vertically slowly varying) buoyancy frequency, *N*, below the mixed layer base. This is in contrast to the assumption that the mixed layer is not or very weakly stratified while the region below is. However, Sutherland (2016) and Pütz et al. (2019) showed that near-inertial waves with horizontal wavelengths much larger than the height of a vertically mixed region have a transmission coefficient through that region near 1. That is, in the long-wave limit, such as that assumed in the hybrid solution (Olbers et al. 2020), gravity waves propagate in the vertical approximately undisturbed by a discontinuity of the stratification. Nonetheless, as it will be shown below, the resulting energy flux estimates are sensitive to the estimate of the stratification below the mixed layer. Following Olbers et al. (2020), we write the Fourier transformed local vertical energy flux $\psi _{_{\text {F}}}$ and its integral ${\varPsi }_{_{\text {F}}}$ as follows:

9 $$ \begin{array}{@{}rcl@{}} \psi_{_{\text{F}}} &=& -\rho \frac{F(\sigma)}{{k}}  \left( \hat{w}({\boldsymbol{k}}, D, \sigma) \hat{w}^{*}({\boldsymbol{k}}, D, \sigma)\right) , \end{array} $$

10 $$ \begin{array}{@{}rcl@{}} {\varPsi}_{_{\text{F}}} &=& -\rho \underset{\mathbb{R}^{2}}{\iint} {{\int}_{f}^{N}} \frac{F(\sigma)}{{k}}  \left( \hat{w}({\boldsymbol{k}}, D, \sigma) \hat{w}^{*}({\boldsymbol{k}}, D, \sigma) \right)  d\sigma  d{\boldsymbol{k}}.\\ \end{array} $$

Here, the asterisk denotes the complex conjugate so that $\left (\hat {w}({\boldsymbol {k}}, D, \sigma ) \hat {w}^{*}({\boldsymbol {k}}, D, \sigma ) \right )$ is the power spectral density of the vertical velocity at the mixed layer base. *N* is the local buoyancy frequency below the mixed layer, and the Coriolis frequency *f* is assumed to be locally constant. The factor $F(\sigma )=\sigma ^{-1} (N^{2}- \sigma ^{2})^{\frac {1}{2}} (\sigma ^{2} - f^{2})^{\frac {1}{2}}$ emerges from the multiplication of the vertical group velocity with the total energy density. The vertical velocity is computed from the slab model results using Eq. 8.

To simplify and speed up the calculation, we assume a dominant wave number *k* with horizontal wave length *L* = 2*π*/*k* in the vertical velocity. Furthermore, we define $\hat {h}(\sigma )=\sqrt {\frac {F(\sigma )}{N-|f|}}$ so that Eq. 9 can be rewritten:

11 $$ \psi_{_{\text{F}}}=-\rho \frac{{L}}{2\pi} (N-|f|) \hat{h}^{2} \left( \hat{w}({\boldsymbol{k}}, D, \sigma) \hat{w}^{*}({\boldsymbol{k}}, D, \sigma) \right). $$

Using Parseval’s theorem for the total energy transfer we deduce the vertical energy flux *ψ* and the total vertical energy transfer *Ψ* in physical space:

12 $$ \begin{array}{@{}rcl@{}} {\psi} &=& -\rho \frac{{L}}{2\pi} (N-|f|) \left|h(t)\ast w(\boldsymbol{x}, D, t)\right|^{2}, \end{array} $$

13 $$ \begin{array}{@{}rcl@{}} {\varPsi} &=& \underset{\mathbb{R}^{2}}{\iint} \int {\psi}  dt d\boldsymbol{x}. \end{array} $$

where *h* is the inverse Fourier transform of $\hat {h}$ and *d****x*** represents the horizontal surface element. The asterisk ∗ denotes the convolution in time. Note that $\hat {h}(\sigma )$ is a normalized window within the interval $\left (|f|,N\right )$. The expression *h*(*t*) ∗ *w*(***x***, *D*, *t*) therefore implicitly depends on the integration interval $\left (|f|,N\right )$ as well as the shape of the window $\hat {h}(\sigma )$. However, the effects are negligible given the assumptions |*f*|≪ *N* in combination with a red spectrum of the wind stress. Thus, the vertical energy flux *ψ* is approximately linearly dependent on the buoyancy frequency *N*. The energy flux estimates are therefore sensitive to the parameter input for *N* and might lead to an overestimation or underestimation when *N* has a corresponding bias. Note that since the dispersion relation for internal gravity waves inherently filters motion with frequencies below the Coriolis frequency, *f*, and above the buoyancy frequency, *N*, we do not pre-process and filter the slab response for the evaluation of the vertical velocity, *w*, at the mixed layer base. For a full derivation with detailed properties, we refer to the companion paper by Olbers et al. (2020).

## Data and input parameters

### Wind stress, mixed layer depth, and buoyancy data

The slab model is forced by the ratio of the local wind stress and the mixed layer depth. In addition, the stratification at the base of the mixed layer is needed to evaluate the energy flux into the interior, *ψ*. The wind stress data should have a temporal resolution of at least 1h to ensure reasonable coverage of the previously described resonance at *σ* = −*f* (Rimac et al. 2013). Moreover a spatial resolution higher than 50km is necessary since the horizontal resolution of the vertical velocity puts limits on the estimate of dominant horizontal wave numbers with expected wavelengths of the order 100km.

Wind stress fields from the NCEP-CFSR reanalysis (Saha et al. 2010) were chosen to force the slab model. A horizontal resolution of 0.3° with hourly values ensures computation of horizontal gradients and dominant wave numbers as well as the full coverage of the resonance peak in the slab equation (4). The years 1989 and 1996 have been chosen as examples for conditions with high and low NAO index respectively (Hurrell and Deser 2009). Positive NAO phases are characterized by stronger wind stresses in the North Atlantic between 40° N and 60° N. Thus, a stronger ocean response is expected. This difference serves as benchmark for the importance of the wind stress magnitude on ratios of energy fluxes at these latitudes.

Since the energy flux through the ocean surface, $\phi _{_{\text {NI}}}$, is proportional to the horizontal velocity in the mixed layer which is inversely proportional to the mixed layer depth (7) and (4), the choice for a data set of the mixed layer depth *D* is crucial for energy flux considerations. Given the existence of unphysically deep, or missing, values in the NCEP-CFSR mixed layer data set, we refrain from using the same product that we use for the atmospheric forcing. Instead, the MIMOC climatology (Schmidtko et al. 2013) was used. In accordance with the approximation of a slowly varying mixed layer depth, high-frequency responses like diurnal cycles and individual entrainment events are not represented. As these effects are neglected, this study focuses on spatial averages and seasonal cycles. We take our definition of *D* from the MIMOC climatology, which makes use of an objective mixed layer pressure algorithm based on density profiles (Holte and Talley 2009) and delivers monthly averaged values with 0.5° horizontal resolution. Moreover, it preserves synoptic features and the influence of the bathymetry while filtering transient phenomena like eddies and internal gravity waves. The data points were set to mid-month dates and linearly interpolated in time and space to obtain hourly time series in the slab model calculation. This avoids strong ringing effects in the corresponding Fourier transforms in time and minimizes its noise. However, the linear interpolation of monthly averages weakens the seasonal cycle and adds a bias to the annual mean of the near-inertial wind power, $\phi _{_{\text {NI}}}$.

In accordance with the mixed layer depth data, the MIMOC data set provides the corresponding optimally interpolated conservative temperature and absolute salinity profiles. They have vertical resolution from 5 to 50dbar increasing with depth. The buoyancy frequency is calculated using finite differences and the Thermodynamic Equation of Seawater 2010 (McDougall and Barker 2011). Overturns within the mixed layer may lead to negative values for the linearly interpolated square of the buoyancy frequency at the mixed layer depth. To get the stratification associated to the mixed layer base, we use a one-sided nearest neighbor interpolation where necessary. It should be stressed that as the radiation energy flux, *ψ*, linearly depends on the buoyancy frequency, *N*, the results strongly depend on how *N* is determined. The use of climatologic values has therefore a twofold advantage. First, it is in accordance with the climatologic mixed layer depth; and second, it corresponds to the WKBJ assumptions of the hybrid slab model where *N* is a slowly varying parameter. We therefore choose a method as robust as possible; however, it remains an important source of uncertainty.

### The damping parameter *r* and the horizontal wavelength *L*

In order to evaluate the damping parameter *r*, 61 moored time series of wind and surface currents were analyzed following the procedure of Alford (2001). However, the results showed a large spread in optimal damping parameters. Additionally, damping time scales evaluated from drifter trajectories (Park et al. 2009) suggested an almost constant parameter throughout the North Atlantic. Hence, a constant damping time *r*^− 1^ = 5days was chosen. More details on the procedure and comparison of the data sets can be found in Appendix B.

The dominant wavelength was estimated from boxed Fourier transforms of the simulated vertical velocity. These were done at 45 locations in the North Atlantic with latitudes ranging from 15° N to 45° N. From the results, a linear parameterization depending on the latitude was derived for both years. The results vary between approximately 250 km and 500 km (Table 1). This is within the range of results found with general circulation models (Simmons and Alford 2012; Rimac et al. 2016) and in surface drifter tracks (Park et al. 2009). Details on the boxed Fourier transform analysis are described in Appendix B.

**Table 1:** Parameterization for horizontal wavelength with function *L* = *αlat* + *β*

Year	$\alpha (\frac {\text {km}}{{~}^{\circ }\text {lat}}) $	*β*(km)
1989	4.1 ± 0.8	231.0 ± 31.2
1996	2.9 ± 0.7	277.3 ± 24.6

The errors indicate double standard deviations from linear fits

## Results

**Fig. 1:**
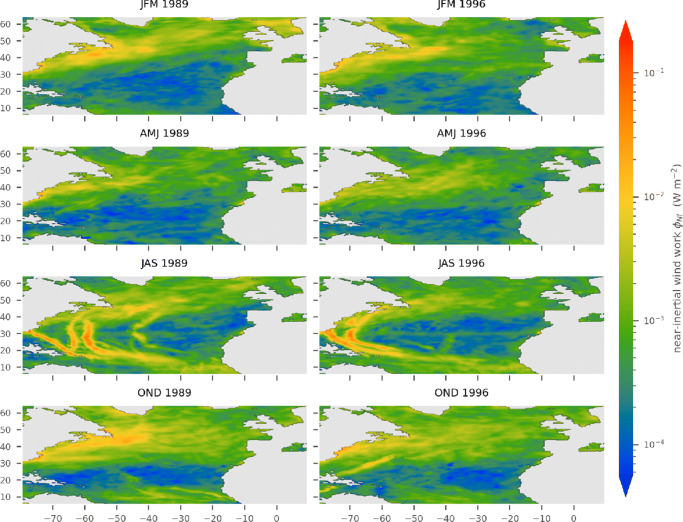
Seasonally averaged near-inertial wind power $\phi _{_{\text {NI}}}$ for 1989 (left) and 1996 (right) computed with the classical slab model

**Fig. 2:**
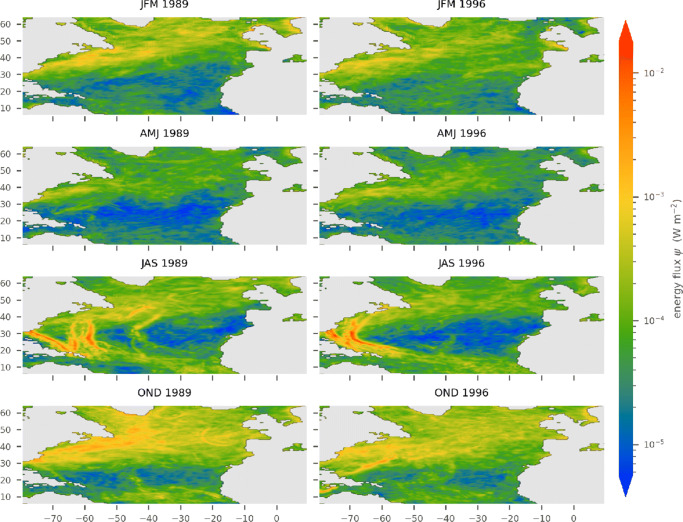
Seasonally averaged energy flux from the mixed layer into the internal wave field *ψ* for 1989 (left) and 1996 (right) computed with the hybrid extension

In general, the two years 1989 and 1996 show similar spatial and temporal patterns in both the near-inertial wind power, $\phi _{_{\text {NI}}}$, and the vertical energy fluxes from the mixed layer into the internal wave field, *ψ* (Figs. 1 and 2). The amplitude of ${\varPhi }_{_{\text {NI}}}$ is about one order of magnitude larger compared with *ψ*. Spatial patterns tend to be more confined in *ψ*. Narrow tracks and rather sharp horizontal gradients are visible (Fig. 2).

### Spatial patterns

The zonal homogeneity of the fluxes in Figs. 1 and 2 (hurricane tracks excluded) suggests that a zonal mean perspective is useful: latitude bands will be referred to as the tropics (south of 20° N), subtropics (between 20° N and 40° N), and mid-latitudes to subpolar regions (north of 40° N).

**Fig. 3:**
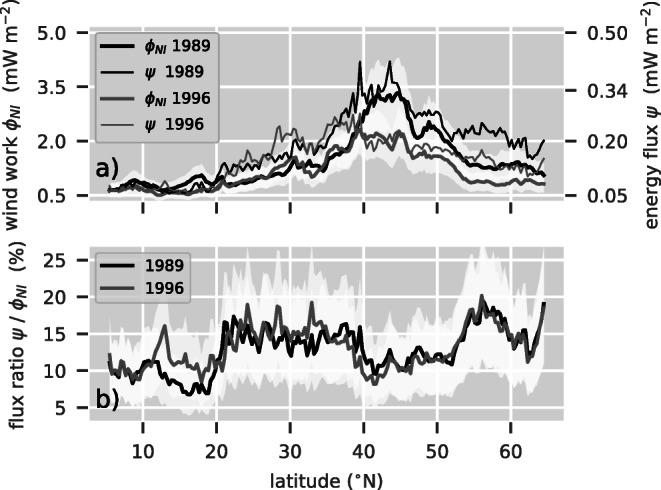
**a** Zonally averaged near-inertial wind power $\phi _{_{\text {NI}}}$ (thick lines) and energy flux *ψ* (thin lines). Note that the energy fluxes *ψ* correspond to the right axis with scaled magnitude. **b** Ratios of fluxes from upper panel. The white shadings depict the standard error of the wind power (a) and the energy flux ratio (b) (cf. Section 55.3)

The tropics are characterized by a low to moderate near-inertial wind power, $\phi _{_{\text {NI}}}$, with valuves up to 1 mWm^− 2^ (Fig. 1). Also, *ψ* has low magnitude with values around 0.05 mWm^− 2^ (Fig. 2). Here, values from both years are similar with the near-inertial wind work, ${\varPhi }_{_{\text {NI}}}$, being slightly elevated in 1989 (Figs. 1 and 3). In the subtropics, both energy fluxes are lowest compared with those of all other regions in the North Atlantic (Figs. 1 and 2). However, within this regime, hurricanes and tropical depressions have the highest intensity; thus, the strongest energy fluxes are observed. The energy fluxes $\phi _{_{\text {NI}}}$ and *ψ* have values of up to 100 mWm^− 2^ and 10mWm^− 2^, respectively (Fig. 1 and 2). Like in the tropics, the years 1989 and 1996 are similar in extent and magnitude of patterns of both energy fluxes beyond the hurricane tracks. The mid-latitudes are dominated by large energy fluxes associated with synoptic-scale storms. In all seasons, the near-inertial wind power, $\phi _{_{\text {NI}}}$, is relatively high (compared with other latitudes). Annual and area averages range from 2 to 4 mWm^− 2^ (Fig. 3) and large regions between 40° N and 50° N and west of 40°W have seasonal means that exceed 10mW m^− 2^ (Fig. 1). The positive NAO year (1989) exhibits larger amplitudes and stronger gradients. *ψ* shows the same features with amplitudes being an order of magnitude smaller (Fig. 2). In subpolar regions north of approximately 55° N, both fluxes visibly decrease.

The different meridional regimes and differences between the two years can be summarized using zonal means (Fig. 3a). Higher amplitudes in both energy fluxes in the mid-latitudes and subpolar regime for 1989 compared with 1996 are now quantitatively visible. Stronger winds north of 40° N in 1989 lead to the energy fluxes increasing by $\sim 50 \%$. Despite the differences between the 2 years, the energy flux ratios, $\psi /{\varPhi }_{_{\text {NI}}}$, show a similar structure (Fig. 3b). Every regime has a typical ratio. Whereas the tropics and subtropics have a rather constant ratio of around 10% and 15%, respectively, the mid-latitudes and subpolar region have a pronounced increase from 10% at 40° N to approximately 18% at 58° N. This is where mixed layers depths are deepest. It then drops to 12% at 63° N.

### Seasonal variation

**Fig. 4:**
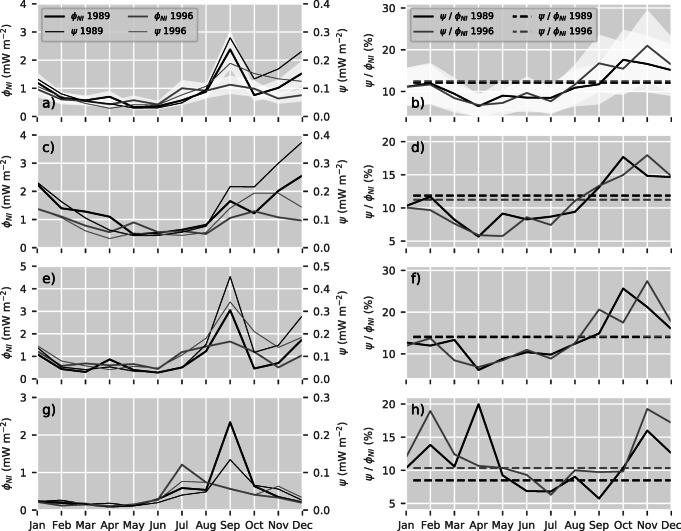
Monthly near-inertial wind power $\phi _{_{\text {NI}}}$ (thick lines) and energy fluxes *ψ* (thin lines) averaged over the whole domain (**a**), the mid-latitudes (**c**), the subtropics (**e**), and the tropics (**g**). Note that the energy fluxes *ψ* correspond to the right axis with scaled magnitude. Additionally, ratios of monthly fluxes (solid) and ratios of average fluxes (dashed) for the whole domain (**b**), the mid-latitudes (**d**), the subtropics (**f**), and the tropics (**h**) are plotted. The white shadings show the standard error of the average near-inertial wind power (**a**) and the energy flux ratio (**b**) (cf. Section 5.3)

The spatial patterns associated to the meridional regimes follow distinct seasonal changes (Figs. 1 and 2). Tropical trade winds with low variability lead to a generally low amplitude in the annual cycle of the energy fluxes ${\varPhi }_{_{\text {NI}}}$ and *ψ* in the tropics (Fig. 4g). Additionally, there is a characteristic rise in energy fluxes during the hurricane season, narrower and stronger in 1989 than in 1996. Energy fluxes in the mid-latitudes are characterized by dominant annual cycles with elevated levels during autumn and winter (Fig. 4c). During 1989, the annual cycle is more pronounced and the average fluxes are larger up to a factor of 2 compared with 1996. In the subtropics, the seasonal variations of ${\varPhi }_{_{\text {NI}}}$ and *ψ* comprise characteristics from both the tropics and mid-latitude regions (Fig. 4e). They show generally low values, a rise during autumn and winter, and the distinctive peaks associated to hurricanes. The annual cycles for the whole North Atlantic (Fig. 4a) are similar to those observed by Alford and Whitmont (2007).

Even though the 2 years exhibit very distinct annual cycles, the ratios of monthly energy fluxes, ${\psi } / \phi _{_{\text {NI}}}$, agree closely in all meridional regimes (Figs. 4b, d, f, and h). They show a minimum between March and May and a maximum between September and October. This ratio ranges from 6 to 20% in the tropics (Fig. 4h), from 6 to 27% in the subtropics (Fig. 4f), and from 6 to 18% in the mid-latitudes and subpolar regions (Fig. 4d). For the whole North Atlantic, it varies between 6 and 21%. The ratios of the annual average energy fluxes in the different latitude bands reflect the meridional structure explored in Fig. 3b. They are 8% for 1989 and 10% for 1996 in the tropics (Fig. 4h), 14% for both years in the subtropics (Fig. 4f), and 12% and 11% for 1989 and 1996 in the mid-latitudes (Fig. 4d), respectively. Averaging the energy fluxes over the whole model domain and the whole year results in a ratio of 12% for both years (Fig. 4b).

## Discussion

To interpret the results described in the previous section, we first consider the differences between the 2 years as an indicator to the sensitivity of the energy fluxes to the wind stress magnitude. We then analyze the parameters with known dependencies. In particular, the energy flux ratio is linearly dependent on the mixed layer depth *D*, the sub mixed layer buoyancy frequency *N*, and the horizontal length scale *L* (Eqs. 7 and 12). Additionally, the influence of the spatial and temporal structure of the wind stress field are analyzed with the aid of simulations of idealized wind stress fronts.

### The two years

The most important difference between the forcing in the two years considered, 1989 and 1996, is the magnitude of the wind stress in mid-latitude regions between 40° N and 60° N. 1989, the year with positive NAO phase (Hurrell and Deser 2009), exhibits stronger winds and therefore higher wind stress amplitudes in this region. Throughout the winter months, both energy fluxes are dominated by mid-latitude storms (Figs. 1 and 2). Consequently, the near-inertial wind power, NI, and the energy flux into the internal wave field, *ψ*, in the respective year are increased correspondingly. This is particularly visible in the zonally and temporally averaged energy fluxes (Fig. 3). North of 40° N, the energy flux associated to the positive NAO phase (1989) is elevated by approximately 50% with respect to the negative phase in 1996. Also, the annual cycle of 1989 is elevated in January, November, and December relative to the annual cycle of 1996 (Fig. 4). In addition, the annual cycles of both years differ with respect to the hurricane season from July through October. Isolated storms injecting large amounts of energy lead to an increase of the energy fluxes in the months of their occurrence. Whereas the hurricane season is reflected by the sharp peak in September of the year 1989 (Fig. 4a), it manifests as a rather broad elevation in the months July through October in 1996.

Even though both energy fluxes substantially differ in magnitude for the two years, the ratios of ${\psi } / \phi _{_{\text {NI}}}$ are remarkably similar (Figs. 3b and 4b). Whereas the agreement in the ratios of the temporally and zonally averaged energy fluxes can partially be explained by the parameter dependencies, the annual cycles do not follow the annual cycles of the spatially averaged input parameters, i.e., the mixed layer depth and the sub mixed layer buoyancy frequency.

### Parameter dependencies

#### Mixed layer depth

**Fig. 5:**
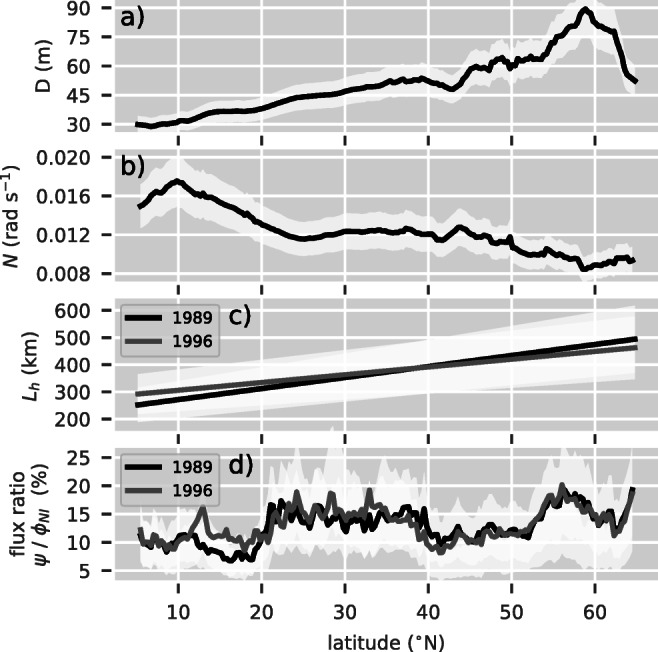
Zonal averages of **a** mixed layer depth; **b** difference between sub mixed layer buoyancy and Coriolis frequency; **c** horizontal length scales *L*; and **d** ratios of yearly averaged out- to influxes. The white shadings represent the standard error of the corresponding parameter (**a**–**c**) and the energy flux ratio (**d**) (cf. Section 55.3)

The near-inertial wind power, $\phi _{_{\text {NI}}}$, is directly proportional to the depth averaged velocity and thus inversely proportional to the mixed layer depth. The zonal average of the climatological mixed layer depth is shallow around 30m at 5° N, increases almost linearly to approximately 65m at 54° N, and peaks at values of around 90m near 59° N. North of the maximum, it drops to 50m at 65° N (Fig. 5a). A similar pattern is observed in the low values of the flux ratios in subtropical regions and the strong transition at 53°N with the subsequent peak at approximately 58°N. This peak in the mixed layers is associated with the strong winter buoyancy loss that drives deep convection in the sub-polar gyre (de Boyer Montégut et al. 2004). Also, note that the zonally averaged NI does not reflect an inverse behavior since the dependence on the wind stress magnitude masks the effect.

The slab model assumes a depth *D*(*t*) at which an internal Reynolds stress, *τ*, equaling the wind stress at the surface, vanishes. However, this depth is not unique as the wind associated internal stresses may be zero at any depth below the assumed minimum value. The additional assumption that this depth corresponds to the mixed layer depth is only true if the mixed layer has been mixed solely by dissipation of wind work. For instance during convection events, the zero-stress depth associated with the wind stress may be only a fraction of the mixed layer depth. If the zero-stress level is overestimated by approximating it with the mixed layer depth, the wind power, $\phi _{_{\text {NI}}}$, is underestimated and the energy flux ratio is overestimated accordingly. This is most likely the case for large areas in the North Atlantic where deep mixed layers are common throughout the winter. On the other hand, some observations have been reported that implied a depth penetration of the wind induced shear below the mixed layer depth (Dohan and Davis 2011; Forryan et al. 2015). Instead of eroding the pycnocline, the internal stress resulted in the broadening of a transition layer and consequently in the reduction of the mixed layer depth. Hence, the assumption that the mixed layer depth is equal to the zero stress level may lead to an over- or underestimation of the wind power in the slab model. The direction of the error is related to the difference between the mixed layer depth and the actual zero stress level. Here, we recall that the vertical velocity at the mixed layer base is independent of the mixed layer depth. Thus, even though $\phi _{_{\text {NI}}}$ scales with the inverse mixed layer depth, this effect cancels in the hybrid extension for the calculation of the energy flux into the internal wave field, *ψ*, but remains present in all energy flux ratios ${\psi } / \phi _{_{\text {NI}}}$. Therefore, the interpretation of the elevated ratios of zonally averaged energy fluxes around 58° N is difficult and possibly an artifact related to deep convection areas.

#### Sub mixed layer buoyancy frequency

The ratio of energy fluxes is approximately linearly dependent on the buoyancy frequency *N* (12). Its zonal average peaks at 18 ⋅ 10^− 3^rad s^− 1^ at 10° N and decreases to 9 ⋅ 10^− 3^rads^− 1^, a relative decrease of 50% (Fig. 5b). We observe that the energy flux ratio does not exhibit any corresponding behavior, presumably because the effect is compensated by the relative increase of the mixed layer depth and the horizontal length scale (see below).

#### Horizontal length scale

*L* is parameterized with a linear increase of values from around 275 km at 5° N to approximately 475 km at 65° N (Fig. 5c). This relative increase is similar to the relative decrease in the buoyancy frequency *N*. Thus, the effects compensate each other and are barely visible in both *ψ* as well as the energy flux ratio *ψ*/NI.

However, there are several sources of uncertainty associated with this parameter. By considering dominant wavelengths, we make use of effective length scales computed from horizontal wave number distributions. In general, we have little knowledge about these forced horizontal wave number spectra. They depend very much on the generation mechanism of the induced vertical velocity and thus on the temporal and spatial structure of the wind. Also, the parameterization from the boxed Fourier transform analysis is problematic. With residues up to 25% in zonal averages, it might have larger deviations from local values. However, we find our values within the range of typical values of *L* from 100 to 1000km (Rimac et al. 2016; Simmons and Alford 2012) and include the shortcomings in our error estimates (cf. Section 5.3 and Appendix B).

### Errors of parameters and energy fluxes

In accordance with the underlying assumptions of the slab model (Section 2), we choose climatologic input for the mixed layer depth, *D*, and the associated buoyancy frequency, *N* (cf. Section 3). These slowly varying parameters have monthly variability but neglect diurnal cycles, individual entrainment events, and inter-annual differences. Also, being global climatologic data sets, they are subject to spatial interpolation and smoothing (Schmidtko et al. 2013). We thus estimate the relative standard errors in both the mixed layer depth, *D*, as well as the sub mixed layer buoyancy frequency, *N*, to be 15%.

In order to choose the damping parameter, *r*, we conducted model-buoy comparisons and compared our results with results obtained from drifter trajectories (Park et al. 2009). In the model-buoy comparisons, we found a large spread of values and thus large standard deviations in the corresponding average damping parameter (cf. Appendix B). The work of Park et al. (2009) points at a near-constant damping parameter for the North Atlantic around *r*^− 1^ = 5days (Fig. 10). They utilize auto correlation functions of time series of drifter tracks to obtain the decay time scale of the inertial oscillations. These time series contain a series of similar decay patterns so that a bias may be introduced in the auto correlation function due to this self-similarity. Based on these error sources, we estimate the relative standard error of the here constant damping parameter to be 25%.

The linear parameterization for the horizontal length scale was developed using a boxed Fourier transform scheme (cf. Section 3 and Appendix B). The resulting values range between 275 and 475km. This corresponds to the range of values found in the literature from 100 to 1000km (Rimac et al. 2016; Simmons and Alford 2012). However, the linear parameterization leads to deviations from the boxed Fourier transforms analysis up to roughly 25%. To account for these uncertainties, we estimate the relative standard error of *L* to be 25%.

The near-inertial wind power, $\phi _{_{\text {NI}}}$, is linearly dependent on *r* and inversely dependent on *D*. The relative standard error of $\phi _{_{\text {NI}}}$ is therefore equal to 29%, which is equivalent to a relative total error of 58%. As the radiated energy flux, *ψ*, is linearly dependent on *N* and *L* it has the same relative standard error of 29%. Consequently, the energy flux ratio $\psi / {\varPhi }_{_{\text {NI}}}$ has a relative standard and total error of 41% and 82%, respectively.

### Wind field structure

The parameter dependencies and the magnitude of the wind stress fail to explain both the structure of the flux ratios and their similarity between the two years. This suggests that the temporal and spatial structures of the forcing are the major driver for this ratio.

As discussed above, the ratio of energy fluxes linearly depends on the mixed layer depth, the sub mixed layer buoyancy frequency, and the horizontal length scale. Comparing the zonally and temporally averaged energy flux ratios to the equally averaged input parameters, the ratios can only partially be explained by the input parameters (Fig. 3). Thus, we suggest that the spatial and temporal structures of the wind stress field are not only major drivers for wave-like structures at the base of the mixed layer but also significantly influence the portion of energy that is transferred to internal gravity waves. Differences between the observed meteorological regimes are discussed below.

The tropics are generally characterized by rather steady winds with stable direction and low seasonal variability. Only tropical cyclones and hurricanes traveling northward are associated with fast moving gradients and fronts. Quasi-steady structures with temporal variability paired with a strong gradient in the Coriolis frequency suggest that the beta effect contributes to the generation of vertical oscillations. However, we have no direct evidence that the beta effect is important here. The latitude dependency suggests an increase of both energy fluxes and their ratios with lower latitude (cf. discussion below). Instead, the simulation of the North Atlantic shows low energy fluxes *ψ* and ratios of average energy fluxes with low seasonal variability south of 20° N (Figs. 3, 4g and h). During the sporadic occurrence of tropical pressure systems, the energy flux ratios increase (Figs. 1 and 2). We observe a small difference between the two years possibly related to the El Niño Southern Oscillation (Fig. 4g and h). However, the difference is well within the error margins.

**Fig. 6:**
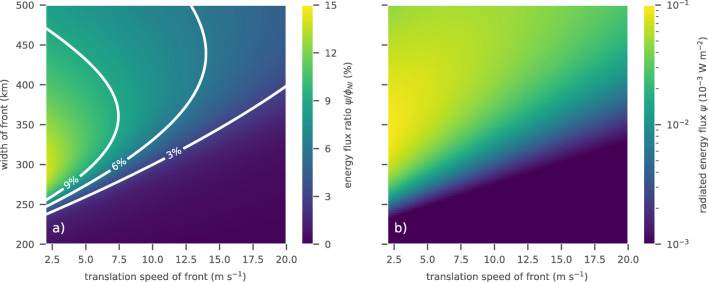
Energy flux ratios associated to idealized Gaussian wind stress fronts translating with the velocity indicated on the *x*-axis. The width of the front is measured by double the standard deviation of the Gaussian and indicated on the *y*-axis

As the wind stress in subtropical regions and the mid-latitudes is characterized by translating structures such as low pressure systems, their offshoots, or hurricanes, we illustrate typical transfer mechanisms using simulations of one-dimensional idealized fronts with Gaussian shape. In particular, we fix the mixed layer depth and the sub-mixed layer buoyancy frequency at 50m and 0.01rads^− 1^, respectively. The horizontal wavelength *L* in the wake of the front is set by its translation speed, $v_{_{atm}}$, and is equal to ${L}=2\pi {v_{_{atm}}} / f$. The width of the Gaussian (twice the standard deviation) and a constant translation speed are free parameters; the maximum amplitude of the wind stress is set to 1N m^− 2^. A more detailed description can be found in Appendix C. Following this simple setup, average energy fluxes and their ratios can be computed using Eqs. 7 and 12. The width of the front is constrained by observations and numerical simulations (Park et al. 2009; Simmons and Alford 2012; Rimac et al. 2016) so that the interval $\left [200{ \text {km}}, 500{ \text {km}}\right ]$ is covered here. A lower bound for the translation speed is set to 2 ms^− 1^, an observed lateral speed of hurricanes (Price et al. 1991). As an upper bound, we choose 20 ms^− 1^, which corresponds to a pressure system crossing the North Atlantic in approximately three days. The resulting distribution of energy flux ratios suggests that fronts with a width of approximately 250 to 300 km and lowest translation speeds are most efficiently injecting energy into the internal wave field with ratios around 14% (Fig. 6a). The ratio decreases for wider or more narrow fronts as well as larger translation speeds. For comparison and as a reference, we additionally show the energy flux into the gravity wave field, *ψ* (Fig. 6b).

Subtropical regions are characterized by the gradient between conditions in tropical and mid-latitude regions. Wind speeds are generally low. The most prominent features are strong tropical storms and fronts associated with pressure systems from mid-latitude regions. Estimating spatial autocorrelation scales of the zonal wind in the North Atlantic and comparing with a Gaussian shape (Park et al. 2009) found standard deviations of the Gaussian structures between 150 and 200km in subtropical regions. That corresponds to the range between 300 and 400km in Fig. 6. Moreover, both offshoots of mid-latitude pressure systems and hurricanes generally move with lateral speeds of a few meters per second. Following the idealized simulations, this suggests high efficiency in energy transfer to the internal gravity wave field with rates above 10% (Fig. 6). Thus, the plateau in the ratios of zonally and temporally averaged energy fluxes for the subtropics may be an effect of the wind stress structure.

In contrast, mid-latitude regions are renowned for large alternating pressure systems with large translation speeds and high seasonal variability. We observe that wind stress structures associated to low pressure systems typically move over the whole North Atlantic within 3 to 7 days. That corresponds to lateral movement speeds between 8 and 20 ms^− 1^. Moreover, the core of a mid-latitude depression is generally significantly larger than both its offshoots and hurricanes. Considering the propagation of idealized fronts, this observation suggests generally lower energy flux ratios compared with the subtropics (Fig. 6). This decrease overlays the increase in mixed layer depth and weakens the corresponding raise of the energy flux ratios (cf. Fig. 5).

A similar setup of an idealized wind stress front can be used to explore the latitude dependency of the energy flux ratio in subtropical and mid-latitude regions. Correspondingly, we fix the mixed layer depth (*D* = 50m), the buoyancy frequency (*N* = 0.01rads^− 1^), the damping time scale (*r*^− 1^ = 5 days), the width of the idealized stress front (2*δ* = 350 km), and the translation speed of the front (${v_{_{atm}}}=11$ ms^− 1^). For a constant Coriolis frequency, *f* = 10^− 4^ rads^− 1^, this corresponds to the previously described idealized simulation with mid-interval parameters (cf. Fig. 6). We now vary the center latitude of a 1000-km track of the front as well as its translation direction. A front moving in east- or westward direction does not experience any gradient in *f*. Note also that the cross-track component of the wind stress is prescribed by the Gaussian shape and the along-track component is zero at all times.

**Fig. 7:**
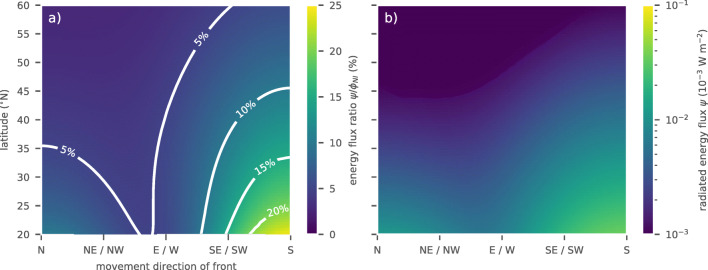
Energy flux ratio ${\psi } / \phi _{_{\text {NI}}}$ for idealized wind stress fronts as function of propagation direction and latitude. The fronts are set with a width of 2*δ* = 350 km and a translation speed of ${v_{_{atm}}}=11{ \text {m s}^{-1}}$

The results indicate that larger Coriolis frequencies or equivalently weaker gradients in the Coriolis frequency lead to lower energy transfer ratios (Fig. 7a). However, the strength of the decrease strongly depends on the movement direction of the front. For northward translating fronts, the energy flux ratio decreases from approximately 10% at 20° N to roughly 3% at 60° N (Fig. 7a). In contrast, southward moving fronts show energy flux ratios from 24.5% at 20° N to 6% at 60° N (Fig. 7a). Thus, southward movement leads to not only higher energy flux ratios but also a stronger decay with latitude. The reason for this behavior is the decay or prolongation of coherent motion in the wake of the front due to the beta effect (not shown). For constant Coriolis frequencies, i.e., east- or westward translation of the front, the energy flux ratio changes linearly from 8.6% at 20° N to 4.2% at 60° N. This effect may be related to the larger window in the frequency response of the ocean at lower latitudes as the interval (|*f*|, *N*) broadens. Again, we show the energy flux into the gravity wave field, *ψ*, for comparison (Fig. 7b).

These findings are in agreement with the results of Zhai (2015). Using the analytic solution of the classical slab model as well as two numerical ocean models, he finds that in the case of a red wind stress spectrum, the excited near-inertial energy is strongly decreasing with latitude. We observe the same behavior in the case of a squared Gaussian wind stress spectrum for both energy fluxes $\phi _{_{\text {NI}}}$ and *ψ* and their ratio ${\psi } / \phi _{_{\text {NI}}}$. However, we find a decrease of the energy flux ratio in the North Atlantic for lower latitudes (Fig. 3). This suggests that the effect may be masked by the parameter dependencies and structural differences in the forcing at different latitudes as highlighted above. Moreover, the idealized model of an advancing wind stress front does not represent latitudes south of 20°N.

### Comparison with previous studies

**Fig. 8:**
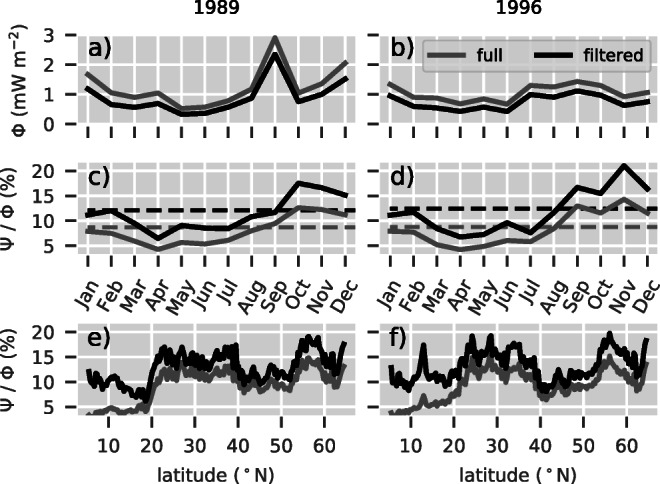
Average wind power (**a**–**b**), ratio of average energy fluxes (**c**–**d**), and ratio of zonally and temporally averaged energy fluxes (**e**–**f**) for the full solution (gray) and filtered slab velocities (black). While the left panels correspond to 1989, the right panels correspond to 1996. Mixed layer currents were bandpass filtered with a Butterworth filter of order 1 in the interval [0.7*f*,1.3*f*]

**Fig. 10:**
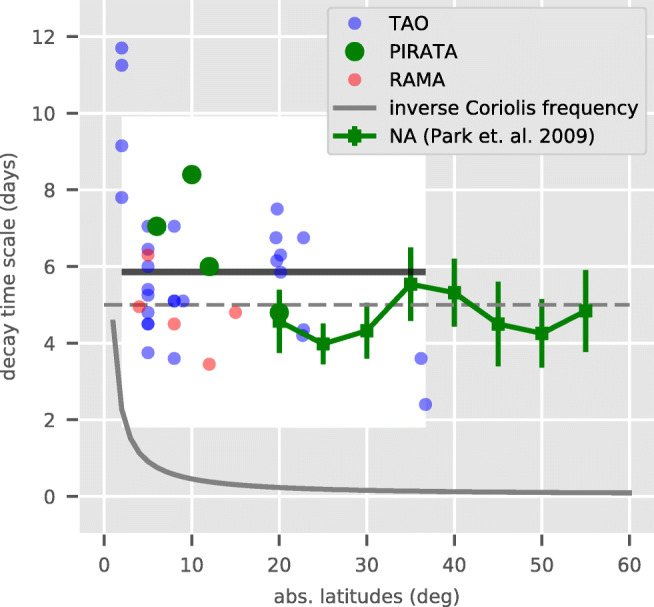
Results from the evaluation procedure, inverse damping parameter *r*^− 1^ plotted against absolute latitude. Round points originate in the present analysis whereas square points are inserted from Park et al. (2009). The black line is the arithmetic mean, the white shading depicts the 95% confidence interval. The dashed and solid gray lines mark a decay time of 5 days and the inverse Coriolis frequency. The results are color coded by ocean basin: red, blue, and green points represent results from the Indian Ocean, the Pacific Oceans, and the North Atlantic, respectively

Three versions of the slab model have been established since its introduction by Pollard and Millard (1970). The original formulation which serves as a mathematical basis of the present study includes the full ocean response to a wind stress damped by a linear friction term and rotated by the Coriolis effect. It has been tested successfully with mooring data (Paduan et al. 1989; Pollard 1980). However, Plueddemann and Farrar (2006) pointed out that the model overestimates the near-inertial kinetic energy in the mixed layer due to missing damping at high frequencies. These results were reproduced by Kilbourne and Girton (2015). All versions of the slab model, including the present hybrid model approach, suffer from the same problem since the assessment of a frequency-dependent damping was not done in detail. Consequently, our estimates of energy flux ratios may be systematically too low. Alford (2003) attempted to modify the equations from Pollard and Millard (1970) in order to introduce a time-dependent friction. However, high-frequency damping remained unchanged. D’Asaro (1985) adapted the formulation by Pollard and Millard and suggested to subtract a time-varying Ekman component. Alford (2001) added a spectral solution scheme to the very same formulation. Here, we follow the commonly used notion of the near-inertial wind power calculated by filtering the mixed layer velocities with a Butterworth band pass filter of order 1 within the interval [0.7*f*,1.3*f*]. Non-resonant sub-inertial spectral components of the excited mixed layer response are therefore effectively removed from the energy flux estimates. The resulting average energy flux ratio of 12% then agrees with ratios estimated by studies based on D’Asaro (1985) and Alford (2001), that is 12–33% as shown by Alford et al. (2012). For comparison, we estimate the total wind power, *ϕ*, as well as the energy flux ratio *ψ*/*ϕ* (Fig. 8). The resulting average total wind power, *ϕ*, is approximately 45% larger (Fig. 8a and b). Consequently, the energy flux ratio, *ψ*/*ϕ*, is smaller by about 3% and results in an average of approximately 9% for both years (Fig. 8c and d). The largest differences can be observed in low latitudes where the sub-inertial components are pronounced due to the comparatively stable trade winds (Fig. 8e and f). Here, the zonally averaged energy flux ratio is smaller by a factor up to approximately 3.5.

Given that the near-inertial wind power, $\phi _{_{\text {NI}}}$, is dependent on the damping parameter, *r*, and the slope of the temporal wind stress spectrum, the energy flux ratio is dependent on the damping parameter *r* in a complex way. Consequently, estimates from different studies utilizing the slab model are difficult to compare when the chosen damping parameters and wind stress spectra vary considerably across the domain.

Since the integrated total kinetic energy is statistically independent of the damping parameter, *r*, and the parameter does not shift the frequency of the excited inertial motion in the mixed layer, an artificial residence time *t*_r_ being equal to the ratio of the average total kinetic energy within the mixed layer, $\iiint \mathcal {E}_{_{\text {kin}}} dV$, and the average energy flux, *ψ*, may be useful for comparison. We find $t_{_{\mathrm {r}}} \approx 31$ days for 1989 and $t_{_{\mathrm {r}}} \approx 29 \text {days}$ for 1996. This roughly corresponds to values found in the literature varying around 20 days (D’Asaro et al. 1995; Levine and Zervakis 1995; Moehlis and Smith 1997). Note that $\mathcal {E}_{_{\text {kin}}}$ represents the time it would take to transfer all the mixed layer kinetic energy into the internal gravity wave field at the average rate internal gravity waves are generated. The values of $t_{_{\text {d}}}\approx 30$ days are thus consistent with an average energy flux ratio of 12% and the prescribed decay of the energy in the mixed layer in the hybrid slab model of $t_{_{\text {d}}} = 1 / 2r = 2.5$ days. Park et al. (2009) showed in their analysis that the linear radiation model introduced by Young and Ben Jelloul (1997) reproduces the decay time scale of surface inertial motions observed by satellite tracked drifters and concluded that the decay time scale corresponds to the radiation time scale of near-inertial waves into the ocean. It is now tempting to conclude that the decay time scale of the surface kinetic energy is generally controlled by wave radiation. However, following our resonance analysis (Appendix Appendix), the largest fraction of energy is located at frequencies smaller than the interval [*f*, *N*] and is therefore not subject to downward wave radiation. Consequently, a decay time scale of the general surface motion being consistent with the internal wave generation time scale allows for the conclusion that the sub-inertial surface response decays on time scales similar or shorter than the wave generation scale. However, no statement about the fraction of energy radiated as internal waves can be made. The dissipation of motion other than the near-inertial amplitude is not discussed by Park et al. (2009). To our knowledge, there is no general discussion on the frequency-dependent dissipation of the surface motion so that a frequency-dependent parameter, *r*(*σ*), could be constructed.

Ratios of average energy fluxes have been quantified using general circulation models (Rimac et al. 2013; Rimac et al. 2016; Furuichi et al. 2008) and direct observations (D’Asaro et al. 1995; Alford et al. 2012) in the past. They range from 11.4% (Rimac et al. 2016) to (37 ± 10)% (D’Asaro et al. 1995). We thus find that our estimate of the average energy flux ratio agrees with estimates from both modeling approaches (e.g., Rimac et al., 2016), as well as direct observations (e.g., Alford et al., 2012). Moreover, we find that our estimates of the near-inertial wind power agree with the recent observations by Liu et al. (2019) in both patterns and magnitudes.

It has been shown that the vertical propagation of near-inertial oscillations depends on the horizontal vorticity (Kunze 1985; Young and Ben Jelloul 1997; Elipot et al. 2010; Whitt and Thomas 2015). That is, a sheared mean flow may modulate the wave numbers and thus vertical group velocities of the generated wave motion. Since the slab model is a result of the linearization of the Boussinesq momentum equation around a zero mean flow, it does not contain the corresponding modulation effects. This adds a bias compared with measurements and eddy resolving models.

Despite its simplified nature, the hybrid slab model was successfully used for qualitative comparisons with moored observations by Köhler et al. (2018). An analytic analysis of the energy transfer spectra compared with an extension directly including wave radiation is presented by Olbers et al. (2020). They extend the here presented approach by setting the Rayleigh damping parameter to zero and instead integrating the internal stresses in the mixed layer to determine the dissipation within the slab. The surface slab is then coupled to the locally excited internal gravity wave field below the mixed layer base by finding an expression for the local pressure disturbance.

## Summary and conclusions

A slab model following Pollard and Millard (1970) has been set up. NCEP-CFSR reanalysis fields of wind stress with 0.3° lateral and 1-h temporal resolution and the MIMOC climatology for mixed layer depth were used to force the model. It was then extended by a formulation for the downward energy flux from the mixed layer base inferred from vertical oscillations on the mixed layer base (Olbers et al. 2012). Simulations of the North Atlantic and idealized wind stress fronts were performed. The area of consideration is affected by meteorological forcing on various time and spatial scales. Different NAO conditions were compared. Buoyancy frequencies at the mixed layer base were estimated using interpolation of the MIMOC absolute salinity and conserved temperature fields. This way we were able to isolate effects based on differences in both magnitude and structure of the various input parameters and forcing data.

Spectral considerations reveal that the amplitude and width of the resonance and therefore the ocean response in the slab model depend on the damping parameter *r*. However, for physical damping time scales of several days, the resonance peak is very narrow while the flanks are non-zero. Thus, the spectral behavior of the ocean response is determined by the spectral power of the wind stress beyond the resonance frequency − *f* and resonance plays a role only in the case of narrow wind stress spectra centered at the negative Coriolis frequency. To reduce this dependency, we compute the near-inertial wind power, ${\varPhi }_{_{\text {NI}}}$, making use of a bandpass Butterworth filter of order 1 with the filter interval [0.7*f*,1.3*f*]. For typically pink to red wind stress spectra (Gille 2005; Zhai 2015), the near-inertial wind power is then significantly less sensitive to the damping parameter, *r*. The kinetic energy density, and thus the energy radiated from the mixed layer base, is independent of it. Frequency-independent damping leads to the same ring down time scale across all frequencies. Plueddemann and Farrar (2006) pointed out that this way mixed layer velocities may be systematically overestimated.

We confirm that the wind power $\phi _{_{\text {NI}}}$ is inversely proportional and the ratio of energy fluxes ${\psi } / \phi _{_{\text {NI}}}$ is proportional to the mixed layer depth. However, this dependency is based on the assumption of the equality of the mixed layer depth and the zero-stress level associated with the wind stress. This assumptions may fail e.g. in deep convection areas and lead to lower estimates of wind power and accordingly higher estimates of energy flux ratios. Some observations suggest that the maximum depth of wind penetration may be deeper than the mixed layer depth (Dohan and Davis 2011; Forryan et al. 2015). In this case, the wind power may be overestimated instead. Since the energy radiated from the mixed layer base, *ψ*, is independent of the mixed layer depth, it gives a more robust estimate in that perspective.

The horizontal length scale *L*, or equivalently the horizontal wave vector of the local vertical oscillation, is prescribed by the generation mechanism. It has typical values of 100–1000 km increasing with latitude. In the simulations of the North Atlantic, we parameterized *L* with the aid of boxed Fourier transform analysis. For idealized simulations with special temporal and spatial structures of the wind forcing it can be approximated by analytic expressions. Nevertheless, the length scale is one of the most important sources of uncertainty.

The radiated energy flux, *ψ*, linearly depends on the buoyancy frequency *N*. The stratification is stronger near the equator and decreases with latitude. However, any effects based on this dependency are masked by the increase in horizontal length scale and mixed layer depth and are therefore not observed in zonally averaged energy fluxes of the North Atlantic. This shows that even though the choice for a climatologic buoyancy frequency is robust and in accordance with the assumptions of the model, it remains an important source of uncertainty in the present approach.

In general, simulations of the North Atlantic only partially follow the described dependencies on input parameters. Ratios of zonally averaged energy fluxes show an increase with latitude similar to the horizontal length scale *L* and the mixed layer depth. This trend is partially compensated by the decrease of stratification with latitude. Moreover, the ratios of zonally averaged energy fluxes indicate several regimes of energy transfer in terms of latitude bands that are not present in the input parameters. In particular, the ratios of zonally averaged energy fluxes have values around 10% in the tropics, and show increased energy transfer ratios of about 15% in the subtropics, and values around 10% north of 40° N. A peak reaching up to 18% around 58° N may be related to deep convection areas. It remains unclear whether this feature is an artifact related to the mixed layer depth data. The ratio of average energy fluxes in the whole domain average to 12%. The near-inertial wind power, ${\varPhi }_{_{\text {NI}}}$, and the energy radiated, *ψ*, themselves are mainly dominated by the magnitude of the wind stress. The wind stress amplitude transfers its seasonal and spatial patterns to both energy fluxes. This can be seen in the zonally averaged energy fluxes from the two years. North of 40° N, fluxes are enhanced by around 50% during the positive NAO phase in 1989. In contrast, the ratios of zonally averaged energy fluxes are independent of the wind stress magnitude. Their very good agreement between the two years suggests importance of the wind field structure.

The tropics (south of 20° N) are determined by rather steady structures with low variability. We observe an increase of the total wind power, *ϕ*, with lower latitudes related to the strength of the trade winds and the latitude dependency of the model. In contrast, the filtered near-inertial wind power, $\phi _{_{\text {NI}}}$, is effectively independent of the low frequency wind stress and therefore significantly smaller. Consequently, the energy flux ratio, ${\psi } / \phi _{_{\text {NI}}}$, is approximately constant in this region while the total energy radiated downward is small in comparison with that of the other regions. Subtropical regions (20° N–40° N) mark the transition between tropical and mid-latitude climates. Slowly advancing fronts associated with offshoots of mid-latitude pressure systems and isolated hurricane tracks dominate the energy fluxes. In contrast, the mid-latitude and subpolar energy fluxes are determined by larger and fast moving pressure systems with high variability. Simulations of idealized Gaussian wind stress fronts with given width and translation speed result in higher energy flux ratios for conditions comparable with subpolar regions and lower energy flux ratios for conditions of the mid-latitude and subpolar North Atlantic. Moreover, they show an increase of both energy fluxes and their ratio with lower latitudes. However, this rise is small for fronts moving in an east- or westward direction. It is concluded that the structure of the wind stress significantly shapes the portion of the wind work that is radiated as internal gravity waves.

In summary, the major advantage of the hybrid solution is the resolution of generation mechanisms of internal gravity waves with a direct quantification of energy radiated from the mixed layer base into the internal wave field. Errors are introduced by the usage of climatological input and the estimate of a dominant horizontal wavelength but also by a frequency-independent artificial damping. These error sources remain to be reduced to make quantitative statements more reliable.

## Figures and Tables

**Fig. 9:**
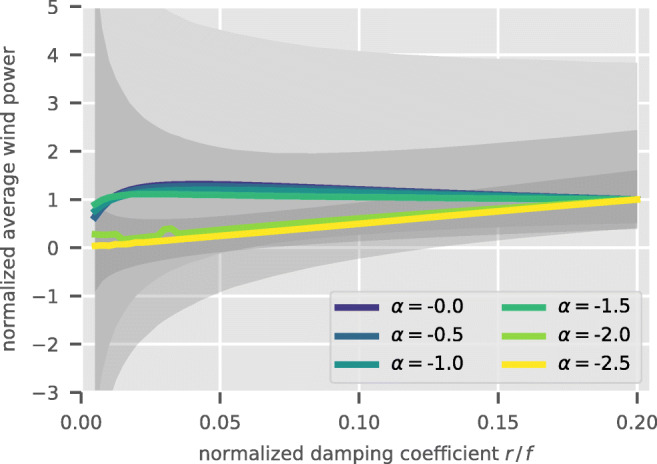
Mean near-inertial wind power $\phi _{_{\text {NI}}}$ averaged over an ensemble of 100,000 realizations of the slab model response to a normalized random wind stress with a given spectral slope *α* in dependency of the damping parameter, *r*. Whereas the solid lines show the maximum probability from skew-normal distribution fits, the gray shading depicts the 5% probability boundary of the ensemble. The model parameters are *f* = 10^− 4^rad / s, *ρ* = 1027kg / m^3^, and *D* = 50m. For an emphasis on the *r*-dependency, all curves and deviations are normalized with the corresponding maximum probability wind power of the at *r* = 0.2*f*

**Fig. 11:**
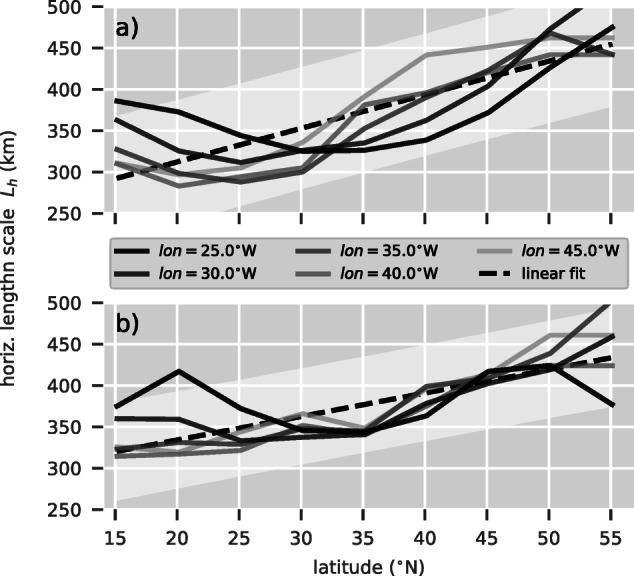
Linear fits to horizontal length scales from boxed Fourier transform spectra for different longitudes for years 1989 (**a**) and 1996 (**b**). White shadings show 95% confidence intervals. Dominant length scales were derived with an asymptotic method to eliminate effects of limited box sizes

## Appendix A: Basic properties of the classical slab model

The governing equations of the classical slab model (Pollard 1970) are given by:

14 $$ {\frac{\partial}{\partial t}} ({u_{_{\text{ML}}}} + i{v_{_{\text{ML}}}}) + (if + r)({u_{_{\text{ML}}}} + i{v_{_{\text{ML}}}}) = \frac{\tau}{\rho_{0} D} $$

where *r* is a constant damping parameter and *τ* = *τ*_*x*_ + *i**τ*_*y*_ are the horizontal components of the vertical fluxes in the stress tensor at the surface, the wind stress. The horizontal velocities, $u_{_{\text {ML}}}$ and $v_{_{\text {ML}}}$, represent an average from a depth *D* to the surface such that it is independent of *z*. This system bases on the assumptions of a rigid lid at the surface, a slowly varying mixed layer depth *D*, vanishing horizontal internal stresses at the mixed layer depth, and a vertically nearly uniform response to the wind stress of the fluid within the mixed layer.

For simplicity, we denote the forcing function *ξ* = *τ*/*ρ*_0_*D* and drop the subscripts in the notation. Additionally, we consider the vertical integral of the continuity equation:

15 $$ \begin{array}{@{}rcl@{}} 0 &=& \int\limits_{-D}^{0} \left[ \nabla \cdot \boldsymbol{u} + {\frac{\partial}{\partial z}} w \right]dz \\ &=& \nabla \cdot {\boldsymbol{u}_{_{\text{ML}}}} + ({\boldsymbol{u}_{_{\text{ML}}}}- \boldsymbol{u}|_{z=-D}) \cdot \nabla D + w|_{z=-D}. \end{array} $$

Note that we have used the rigid lid assumption and the vector notation for the vertically averaged mixed layer velocity ${\boldsymbol {u}_{_{\text {ML}}}} = ({u_{_{\text {ML}}}}, {v_{_{\text {ML}}}})$. Using the assumption of a surface slab ocean with a uniform response throughout the mixed layer so that $\boldsymbol {u}_{_{\text {ML}}} = \boldsymbol {u}|_{z=-D}$, the equation reduces to Eq. 8. Thus, under this assumption, one can formulate a prognostic equation for the uniform mixed layer velocity $\boldsymbol {u}_{_{\text {ML}}}$ and derive the corresponding vertical velocity at the mixed layer depth, *w*|_*z*=−*D*_. Alternatively, one can stick to a transport formulation without loss of the covered effects. The vertical velocity at the mixed layer base is then considered a wave fluctuation consistent with linear Boussinesq theory, whereas the mixed layer depth is defined through the discontinuity of the stratification.

For the analysis of the resonance, we take a closer look at the governing equations (Eq. 1) and assume a periodic and monochromatic forcing $\xi _{x} + i\xi _{y} = \xi _{0}\exp (i\omega t)$. Solving the system gives:

16 $$  u + iv = \frac{\xi_{x} + i\xi_{y}}{r + i(f + \omega)}. $$

The corresponding expression for the total wind power *ϕ* and kinetic energy density $\mathcal {E}_{\text { kin}}$ are:

17 $$ \begin{array}{@{}rcl@{}} \phi(t) &=& \rho D \frac{r ({\xi_{x}^{2}} + {\xi_{y}^{2}})}{r^{2} + (f + \omega)^{2}}, \end{array} $$

18 $$ \begin{array}{@{}rcl@{}} {\mathcal{E}_{_{\text{kin}}}}(t) &=& \frac{\rho}{2} \frac{{\xi_{x}^{2}} + {\xi_{y}^{2}}}{r^{2} + (f + \omega)^{2}}, \end{array} $$

Dividing by the square of the wind stress vector yields the transfer functions for the total wind power, *ϕ*, and the kinetic energy density, ${\mathcal {E}_{_{\text {kin}}}}$. Both transfer functions have a resonance peak at the forcing frequency *ω* = −*f*. The resonance peak of $\phi _{_{\text {NI}}}$ scales with 1/*r* and has a full width at half maximum (FWHM) of *r*. Beyond the resonance, it scales with *r*. In contrast, $\mathcal {E}_{_{\text {kin}}}$ peaks at the same frequency but with the scaling *r*^− 2^. However, it is independent of the damping parameter in the limit of large frequencies, i.e., at (*ω* + *f*)^2^ ≈ *ω*^2^ ≫ *r*^2^. Note that this resonance is subject to the amplitude of the forcing at its frequency *ω*. Given the assumption *r* ≪ *f* and the consequently small FWHM, the resonance affects only a small interval in the spectral transfer. A more robust diagnostic with respect to the damping parameter is the near-inertial wind power, $\phi _{_{\text {NI}}}$, defined by the product of the wind stress and a bandpass filtered slab response so that $\phi _{_{\text {NI}}} = \tau _{0x}u_{\tiny \text {NI}} + \tau _{0y}v_{\tiny \text {NI}}$ where the slab response, (*u*_NI_, *v*_NI_), is calculated by bandpass filtering the slab velocities with a Butterworth filter of order 1 with the filter interval [0.7*f*,1.3*f*]. As a simple sensitivity test, we compute the average response of the classical slab model to a population of 100,000 realizations of a normalized random wind stress with a given spectral slope, *α* (Fig. 9). The spectral slope describes the dependency of the power spectral density of the wind stress on the frequency *ω* such that $\hat {\tau }\hat {\tau }^{*} = P(\tau ) \propto \omega ^{\alpha }$.

For spectral slopes *α* ≥− 1.5, the near-inertial wind power, $\phi _{_{\text {NI}}}$, is approximately independent of the damping parameter, *r*. Only for red wind stress spectra with *α* < − 1.5, we find that $\phi _{_{\text {NI}}}$ is linearly dependent on *r*. Hence, for a typically pink to red wind stress spectrum (Gille 2005), the near-inertial wind power is not independent on the damping parameter but significantly less sensitive than the total wind power, which has generally a linear dependency on *r* for negative spectral slopes *α* < 0 (not shown). Note that the variability of the wind power, here indicated by the boundaries of 5% probability (Fig. 9), includes negative values for *α* ≤− 1.5 and small *r*. The reason for this is that sub-inertial wind stress may excite near-inertial motions which in return may be damped by the near-inertial wind stress itself. In our estimates of the near-inertial response of the North Atlantic ocean, we do not observe a corresponding behavior.

Another parameter supplied to the slab model is the mixed layer depth *D*. In particular, the forcing function *ξ* is inversely dependent on *D* (cf. Section 2). Hence, the wind power, ${\varPhi }_{_{\text {NI}}}$, is proportional to *D*^− 1^ and a the kinetic energy density scales with *D*^− 2^.

## Appendix B: Technical details of the hybrid slab model implementation

The implementation of the model follows a complex Fourier transform scheme based on the complex notation of Eq. 4 (D’Asaro 1985). Since the discrete fast Fourier transform on bounded intervals, in our case 1 year, assumes periodicity of the input, transition signals occur after transforming back. To avoid these numerical errors, the untransformed source function is zero padded corresponding to three decay time scales *t*_*d*_ = 1/*r*. That means we virtually switch off the wind stress at the end of the time series and the corresponding response of the horizontal velocity is an oscillating decay $\boldsymbol {u}=\exp (-rt) (u_{0} \cos \limits (ft), v_{0} \sin \limits (-ft))$. This decay is cut off so that time frames match and the best estimates for mixed layer velocities with initial conditions approximately zero are achieved. However, this procedure introduces a small ringing effect at the beginning of the time series, if the wind stress at model time *t* = 0 is non-zero. Vertical velocities were calculated with midpoint divergences according to Eq. 8. The grid has a resolution of 0.3° which corresponds to approximately 33 km at 5° N and 14 km at 65° N.

Above we showed that the near-inertial wind power, $\phi _{_{\text {NI}}}$, albeit less sensitive compared with the total wind power, *ϕ*, depends on the damping parameter *r* for a pink to red wind stress spectrum (cf. Appendix A and Fig. 9). For a consistent choice of the parameter *r*, data from 42 sites with 61 independently measured time series were analyzed. In particular, measured currents below the water surface were compared with modeled currents from measured wind speeds. The data sets were taken from the Global Tropical Moored Buoy Array (GTMBA) (McPhaden 2010), the Woods Hole - Hawaii Ocean Time-series Site (WHOTS) (Karl and Lukas 1996), the Stratus mooring, the Monterey Ocean Observing System (MOOS) (Chaffey et al. 2001), and the ocean station Papa. Available data with both wind and current measurements is sparse for latitudes beyond 20°N. The method closely followed the procedure of Alford (2001). Measured mixed layer currents were correlated with modeled currents from measured winds at the buoy sites. A range of damping parameters was taken into account and the parameters corresponding to the highest correlation coefficient were considered optimal at the buoy position. To guarantee meaningful results, time series with correlation coefficients smaller than 0.5, optimal damping parameters at the boundaries of the considered interval, and damping parameters larger than half the local Coriolis frequency were disregarded. In total, 38 time series remained after all filters were applied (Fig. 10). This way an estimate for the damping parameter was achieved. However, this approach also has a number of problems.

The model does not resolve a variety of physical processes like surface wave dynamics, stirring of the mixed layer, or entrainment at the mixed layer base. These mechanisms may result not only in temporal but also in spatial dependencies. Available data from buoys equipped with instruments measuring both wind and ocean surface currents are sparse and insufficient for evaluating either the spatial or the temporal variability. Evaluations of the damping parameter *r* with many independently measured time series from the same buoy result in a large spread of values. The consequently low precision is reflected in the large standard deviations in Fig. 10. In addition, the model may become less reliable as the stability criterion *r* ≪ *f* is violated. Also, Alford (2003) pointed out that a frequency-dependent damping might be necessary to achieve more realistic modeled ocean currents.

Averaging over time and grouping into ocean basins (Park et al. 2009) showed that drifter tracks in the North Atlantic differ from the other oceans. They observed an almost constant e-fold damping time scale of approximately 5days and thus no dependence on latitude in the North Atlantic. Despite the large spread, our results correspond to their findings within the confidence interval (Fig. 10). Since we do calculations for the North Atlantic only, a constant damping parameter *r* = 1/5days was used. Taking into account the wide spread of the model-mooring comparisons and the potential bias introduced in Park et al. (2009) due to the consideration of autocorrelation functions of self-similar time series, we assume a conservative relative standard error of 25%.

Additionally, the dominant horizontal wave number needs to be identified. The horizontal spectrum of the vertical velocity is evaluated with the aid of boxed Fourier transforms. Square boxes around an array of fixed positions were multiplied by Hamming windows in both meridional and zonal directions. The corresponding meridional and zonal wave number spectra were calculated and averaged over the box. Wave numbers corresponding to the maxima of these spectra were combined to a dominant horizontal wave number 2*π*/*L*. Since this estimate depends on the chosen box size, the analysis was repeated for box lengths between 3 and 30 points. Moreover, it was done for every time step of the year and averaged temporally. In order to evaluate the horizontal length scale of a virtually infinite box, an asymptotic function of the form $A_{i}\arctan (B_{i} d)$ with parameters *A*_*i*_ and *B*_*i*_, and zonal width of the box *d* was fitted to the results. The amplitude *A*_*i*_ divided by $\frac {\pi }{2}$ was then assumed to be the asymptotic dominant length scale. A grid of 5×9 positions between (15°N,45°W) and (55°N,25°W) was taken into account to find a latitude-dependent linear parameterization. The results for 1989 and 1996 are displayed in Table 1 and visualized in Fig. 11. The maximum deviations within the 95% confidence intervals from the linear fits lead to errors up to 26% and 19% for 1989 and 1996, respectively. The difference from our values of *L* compared with values found in other publications from 100 to 1000 km (Rimac et al. 2016; Simmons and Alford 2012) is about a factor of 2. For a rough error estimation, we thus take a corresponding relative standard error of 25%.

## Appendix C: An idealized wind stress front

The influence of the spatial and temporal wind stress structures upon the energy flux ratios is illustrated using simulations of idealized wind stress fronts. In particular, we construct a Gaussian zonal wind stress moving in meridional direction following:

19 $$ \begin{array}{@{}rcl@{}} \xi_{x} &=& \frac{A}{\rho D}\exp\left( -\frac{(y - {v_{_{atm}}} t)^{2}}{2\delta^{2}}\right), \end{array} $$

20 $$ \begin{array}{@{}rcl@{}} \xi_{y}&\equiv& 0, \end{array} $$

with the wind stress amplitude *A*, the translation speed ${v_{_{atm}}}$, and the standard deviation of the Gaussian *δ*. As before, *ρ* and *D* are the reference density and mixed layer depth, respectively. Due to the symmetry, this setup is effectively 1-dimensional and the forcing ***ξ*** depends only on the meridional coordinate *y* and the time *t*. The parameter *δ* characterizes the width of the moving pulse. However, in the main text, the width refers to the length scale 2*δ*. The horizontal length scale is inferred from the phase shift originating in the lateral movement of the front and is $L=2\pi v_{_{atm}} / f$. Using the fixed and varying parameters summarized in Table 2, energy fluxes and their ratios can be computed using Eqs. 7 and 12.

**Table 2:** Parameters used for the simulation of idealized fronts

Parameter	Value	Comment
Fixed parameters		
*A*	1 N m^2^	Wind stress amplitude
*ρ*	1027 kg m^3^	Reference density
*D*	50 m	Mixed layer depth
*f*	10^− 4^ rads^− 1^	Coriolis frequency
*N*	10^− 2^ rads^− 1^	Buoyancy frequency
Varying parameters		
${v_{_{atm}}}$	2–20 m s^− 1^	Lateral speed of the front
*δ*	100–250 km	Half width of front
